# Arctic Coralline Algae Elevate Surface pH and Carbonate in the Dark

**DOI:** 10.3389/fpls.2018.01416

**Published:** 2018-09-25

**Authors:** Laurie C. Hofmann, Kathryn Schoenrock, Dirk de Beer

**Affiliations:** ^1^Max Planck Institute for Marine Microbiology, Microsensor Group, Bremen, Germany; ^2^Department of Geographical and Earth Science, University of Glasgow, Glasgow, United Kingdom

**Keywords:** calcification, carbon concentrating mechanism, carbonate chemistry, light-independent carbon fixation, microenvironment, microsensor, rhodolith

## Abstract

Red coralline algae are projected to be sensitive to ocean acidification, particularly in polar oceans. As important ecosystem engineers, their potential sensitivity has broad implications, and understanding their carbon acquisition mechanisms is necessary for making reliable predictions. Therefore, we investigated the localized carbonate chemistry at the surface of Arctic coralline algae using microsensors. We report for the first time carbonate ion concentration and pH measurements ([CO_3_^2-^]) at and above the algal surface in the microenvironment. We show that surface pH and [CO_3_^2-^] are higher than the bulk seawater in the light, and even after hours of darkness. We further show that three species of Arctic coralline algae have efficient carbon concentrating mechanisms including direct bicarbonate uptake and indirect bicarbonate use via a carbonic anhydrase enzyme. Our results suggest that Arctic corallines have strong biological control over their surface chemistry, where active calcification occurs, and that net dissolution in the dark does not occur. We suggest that the elevated pH and [CO_3_^2-^] in the dark could be explained by a high rate of light independent carbon fixation that reduces respiratory CO_2_ release. This mechanism could provide a potential adaptation to ocean acidification in Arctic coralline algae, which has important implications for future Arctic marine ecosystems.

## Introduction

Red coralline algae play key roles as ecosystem engineers in global oceans (Foster, 2001; Nelson, 2009), and contribute significantly to carbonate deposition (Amado-Filho et al., 2012; Van Der Heijden and Kamenos, 2015). Some coralline algae, particularly crustose coralline algae, have shown sensitivity to ocean acidification in many laboratory and field studies (Hofmann and Bischof, 2014; McCoy and Kamenos, 2015), but it remains to be determined if they will have the capacity to adapt to the pH decrease and associated decrease in carbonate ion saturation states expected in future surface oceans due to higher dissolved CO_2_ concentrations. In the polar oceans, especially the Arctic, where the carbonate buffering capacity is weak and the system is vulnerable to environmental change (Steinacher et al., 2009; Shadwick et al., 2013), coralline algae may be less resistant to ocean acidification than in lower latitudes, particularly during the dark Arctic winters (Büdenbender et al., 2011). However, recent studies have shown that some coralline algae alter their skeletal structure and composition (Nash et al., 2013; Ragazzola et al., 2013, 2016; McCoy and Ragazzola, 2014; Kamenos et al., 2016) under elevated CO_2_, perhaps in an effort to reduce skeletal dissolution, and they can have strong biotic control over calcification and inorganic carbon uptake (Comeau et al., 2013; Hofmann et al., 2016; Cornwall et al., 2017). It is becoming clear that Arctic coralline algae, particularly rhodoliths (free-living coralline red algal nodules), are more abundant than previously believed. For example, the existence of rhodoliths in eastern Greenland was first reported last year, where several rhodolith beds along the eastern Greenlandic coast were found (Jørgensbye and Halfar, 2017). The authors of this study suggest that there are likely many more beds in remote areas that are not close to research stations or settlements. Because rhodolith bed discoveries have been made around the world recently, the current estimates of the contribution of coralline algae to the global carbonate budget are likely severely underestimated, including their contribution to carbon storage (Van Der Heijden and Kamenos, 2015). Therefore, understanding the mechanism of calcification in polar coralline algae is necessary for making reliable predictions and subsequent policy decisions.

Coralline algae deposit high-Mg calcite in their cell walls, which cement filaments of cells together. They occur as either a thin crust on hard substrata (crustose coralline algae, CCA), as free-living rhodoliths, or as a flat thallus with articulated branches. The surface chemistry of dissolved inorganic carbon (DIC) in calcifying algae can be controlled by several processes. Photosynthesis, dominant in the light, leads to an increase of the pH and carbonate ion concentration [CO_3_^2-^] and thus favors calcification, while net respiration has the opposite effect on calcification (McConnaughey, 1989). Calcification induces the liberation of protons (as Ca^2+^ + HCO_3_^-^ → CaCO_3_ + H^+^), and thus buffers the pH shift induced by photosynthesis, while dissolution can buffer the pH decrease by respiration (Gattuso et al., 1999). Active transport, mostly proton pumping or active uptake of HCO_3_^-^ can induce pH shifts that are independent, and often of different time scales, as the metabolic processes (Borowitzka and Larkum, 1976; McConnaughey, 1991; de Beer and Larkum, 2001; Al-Horani et al., 2003). The latter can be considered as expression of biotic control of calcification (Borowitzka and Larkum, 1977). These ion pumps transport HCO_3_^-^ and H^+^ into the tissue in the light (Borowitzka and Larkum, 1987), where the enzyme carbonic anhydrase speeds up the dehydration of HCO_3_^-^ to CO_2_. Due to photosynthesis and the transport of protons away from the surface, the pH at the coralline algal surface (pH_S_) is elevated compared to the bulk seawater pH (pH_B_) (Hurd et al., 2011; Cornwall et al., 2013, 2015; Hofmann et al., 2016), which favors the surface calcification, due to higher CO_3_^2-^ concentration. In the dark, the reverse process occurs, and the protons released are buffered by the CO_3_^2-^ to form HCO_3_^-^ (Comeau et al., 2013). Further evidence of elevated surface pH at the site of calcification in coralline algae has been shown in recent studies using boron isotopes (Cornwall et al., 2017; Donald et al., 2017). However, the dynamics of CO_3_^2-^ and pH in response to light have never been measured at the surface of a coralline alga. Hence, no data on active processes that control the surface chemistry, like light-induced proton pumps, are available. To elucidate the possible presence of such pumps, we measured the chemistry dynamics at the surface in response to illumination, using microsensors to achieve high-spatial resolution. For describing the complete carbonate system at the surface of coralline algae data on at least two parameters of the carbonate system are needed, assuming equilibrium. Therefore, we measured surface [CO_3_^2-^] and pH using microsensors at the surface of polar coralline algae in response to light dynamics. Although these are not the ideal parameters to use for calculating the remaining parameters of the seawater carbonate system, we do so as a first attempt to provide a glimpse of what the surface chemistry of Arctic coralline algae may look like, considering it is not yet possible to measure DIC or total alkalinity (TA) directly at the algal surface. We hypothesized that surface [CO_3_^2-^] would depend on light, being elevated in the light and reduced in the dark.

## Materials and Methods

### Algae Sampling and Maintenance

**FIGURE 1:**
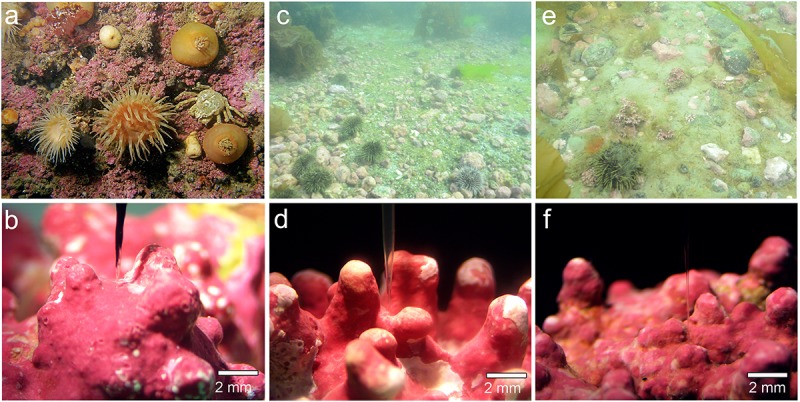
Images from collection sites and examples of specimens investigated: **(a,b)** Kongsfjord, *P. tenue*; **(c,d)** Købbefjord, *L. glaciale*; and **(e,f)** Akia Penninsula, *L. tophiforme*. In **(c)**, high cover of the crustose coralline *Clathromorphum* sp. is apparent. The microsensors used. In **(c,d)**, the microsensor is positioned between nodes/branches. In **(f)**, the microsensor is positioned at the tip of a node. *In situ* photographs are from M. Schwanitz and K. Schoenrock. Microscopic photos taken by L. Hofmann.

Samples of the crustose coralline *Phymatolithon tenue* (Rosenvinge) Düwel & Wegeberg (**Figures 1a,b**) were collected between 10 and 12 m depth at Hansneset, off the southwestern coast of the island Bloomstrand in Kongsfjord, Svalbard, where temperatures range from -2 to 7°C during the year at 20 m depth (Laudien et al., 2014). High sediment deposition from surrounding glaciers results in high freshwater input and high turbidity in Kongsfjord during summer (Hajime and Kudoh, 1997; Hanelt et al., 2001), which strongly attenuates the solar radiation at depth in the fjord (Hanelt et al., 2001). The photosynthetically active radiation (PAR) available at 10 m depth is less than 10% of surface radiation (Cui et al., 2013). Algal samples were transported to the Max Planck Institute for Marine Microbiology (MPIMM), where they were maintained at 4°C in custom-made re-circulating aquaria (20 L) with natural seawater (34 psu) on an 18:6 h day/night cycle at 10 μmol photons m^-2^ s^-1^ for 1 year prior to the experiments. One-third of the seawater in the recirculating tanks was replaced every 2 weeks. Two species of *Lithothamnion* rhodoliths, *L. glaciale* Kjellman (**Figures 1c,d**) and *L. tophiforme* (Esper) Unger (**Figures 1e,f**), were collected using SCUBA from two sites off the southwestern coast of Greenland: Købbe Fjord (*L. glaciale*: 64.14, -51.59) and Akia Penninsula (*L. tophiforme*: 64.193210, -51.908612). Surface currents in the Nuuk region exceed 1 m s^-1^ shortly before and after slack tide, therefore water flow in coralline habitats is due to tidal flux year round. The site at Akia Peninsula experiences flushing from Godthåbsfjord to the marine environment through small channels, while Købbefjord has an influx and outflux of primarily marine water. Currents at the benthos have not been measured due to logistical constraints, but the site at Akia Peninsula specifically has a great deal of sedimentation (**Figure 1e**), indicating flow significantly decreases with depth. The site at Akia Penninsula has high canopy cover of *Saccharina longicruris* (**Figure 1e**), while there is almost no defined canopy cover in Købbefjord, only spotty cover of *Agarum clathratum* (**Figure 1c**). Average mid-day summer light intensities at 10 m depth range measured with an Odyssey PAR logger (Dataflow Systems Ltd, Christchurch, NZ) ranged between 101 and 196 μmol photons m^-2^ s^-1^ at midday. After collection, these specimens were transported to the MPIMM via the University of Glasgow in ambient seawater at 4°C, and then maintained in custom-made recirculating aquaria in the same conditions as Svalbard collections for 9 months prior to the experiments.

### Microsensor Construction

O_2_ microsensors were made and used as described by Revsbech (1989), liquid ion exchange (LIX) pH and CO_3_^2-^ microelectrodes were constructed according to de Beer et al. (2008). The O_2_ optode was calibrated with 0 and 100% air saturated seawater obtained by bubbling with nitrogen gas and compressed air, respectively. All calibrations were conducted at 4°C. The CO_3_^2-^ microelectrode was calibrated following the continuous titration method from Han et al. (2014) using natural seawater. To reduce air exposure, the calibration was conducted in a glass Schott bottle with a Teflon-sealed lid with custom made holes for the pH electrode, microelectrode, and reference electrode. The HCl titration solution was added by inserting a needle syringe through the Teflon membrane. The pH microelectrode was calibrated using pH_NST_ 7.0 and 9.0 buffers (Fluka Analytical, Buchs, Switzerland). The measured pH values were then corrected to the total pH scale by subtracting 0.1 pH units for the seawater chemistry calculations. We acknowledge that certified reference material is preferred for calibrating pH, but the extreme sensitivity of our LIX pH microelectrodes and their incompatibility with some calibration standards did not allow this. Because of the uncertainty associated with NBS buffers and the resulting calculations of the seawater system, we report the profiles of CO_3_^2-^ as ratios rather than absolute concentrations. The oxygen and pH electrodes had average tip diameters between 10 and 20 μm, and therefore minimal influence on the diffusional boundary layer (DBL) of the coralline algae measured.

### Microprofiling

The experimental set-up used for microprofiling was the same described in Hofmann et al. (2016). Seawater was pumped through a plexiglass flow cell (19 cm × 9 cm × 9.5 cm) attached to a temperature-controlled reservoir at approximately 2 cm s^-1^ (judged by particle movement) and passed through a perforated wall at the entrance and exit of the flow chamber to create laminar flow at the algal surface. All experiments were conducted at 7°C. pH and CO_3_^2-^ microelectrodes and an oxygen optode were used to measure depth microprofiles from the surface of the algae into the overlying seawater under both saturating light (100 μmol photons m^-2^ s^-1^) and dark conditions. Microprofiles of the three species were measured under steady state conditions. Up to four individuals from each species were measured on multiple occasions at multiple branch tips (due to variations in DBL thickness depending on morphology, only branch tips were used; see Results below), but complete datasets for all three parameters (pH, O_2_, and CO_3_^2-^) could only be obtained on three replicates of each species due to broken/faulty sensors. In the light, a steady state signal was achieved after several minutes, while a dark adaptation period of at least 30 min was necessary before obtaining a steady signal with no noticeable change. In order to determine when the dark steady state was reached, O_2_, pH, and CO_3_^2-^ at the surface of the algae were monitored continuously overnight (see Light dynamics subsection below).

Microprofiles of O_2_, pH, and CO_3_^2-^ for each individual were obtained by first positioning the sensors at the surface of the algal specimen by hand using a micromanipulator (Pyroscience, Aachen, Germany) with the aid of a stereomicroscope. Using the software Profix (Pyroscience, Aachen, Germany), the microelectrodes were then programed to run four consecutive profiles from the surface of the alga (position 0) into the overlying water column (position 800 μm) at 50 μm intervals. Microprofiles were conducted first under saturating light and steady state conditions. Microprofiles in the dark were then obtained following a dark adaptation period of at least 30 min, after which there was no noticeable change in the sensor signal. Because light and dark profiles were measured at the same location on the same individual at different time points, light was treated as a repeated factor with two levels (light, dark) for statistical analysis in this case (see below). Oxygen fluxes were calculated from the concentration profiles according to Fick’s Law [see Hofmann et al. (2016)] using the diffusion coefficient 1.34 × 10^-9^ m^2^ s^-1^ for seawater at 7°C and 35‰ salinity. The delta pH was calculated by subtracting the surface pH from the bulk seawater pH (ΔpH = pH_S_ - pH_B_), and surface ratios of CO_3_^2-^ were calculated by dividing the surface concentration by the bulk seawater concentration.

Microprofiles of pH at the branch tip and base of rhodoliths showed that branch bases have thicker DBL than branch tips, and consequently, the pH between branches is slightly higher than in the bulk seawater above branch tips (**Supplementary Table S1**). In order to reduce the variation in profile data driven by morphological characteristics, we measured all profiles on branch tips of individual rhodoliths. By standardizing the location of profile measurements, we observed that there was no difference in the boundary layer thickness between the crustose or branched forms investigated (**Supplementary Table S1**).

### Inhibition Experiments

The extracellular carbonic anhydrase inhibitor, acetazolamide (AZ), was used to test for carbonic anhydrase activity and its effect on surface chemistry of one of the species investigated in this study (*L. glaciale*). By inhibiting carbonic anhydrase, we could determine if surface pH was directly coupled to photosynthesis. Prior the addition of the inhibitor, a gross photosynthesis versus irradiance curve was generated to determine limiting and saturating light levels. Gross photosynthesis measurements were conducted using the light–dark shift method (Revsbech and Jørgensen, 1983, 1986), and the preparation of AZ was conducted following the methods by Hofmann et al. (2016). The curve was fit using the nonlinear least squares (nls) command in R with the equation from Eilers and Peeters (1988) that considers potential photoinhibition

$$GP=\frac{1}{{aI}^{2}+bI+c}$$

where GP is gross photosynthesis, I is the irradiance, and a, b, and c are the parameters estimated by the model. After fitting the model to the data, the light saturation point could be estimated by calculating the light intensity at which photosynthesis was saturating. pH profiles and gross photosynthesis were measured at three and five light conditions, respectively, prior to and after the addition of AZ (final concentration 280 μM). The addition of AZ was made to the seawater reservoir while the pH sensor remained on the surface of the alga. Measurements were made after a steady state was reached. Unfortunately, we could only successfully complete this experiment on a single individual, and therefore the figures can be found in the **Supplementary Material**.

### Statistical Analysis

The parameters of the seawater carbonate system were calculated from the pH (total scale) and CO_3_^2-^ concentrations measured at the algal surface and in the bulk seawater using the package seacarb in R. To test for an effect of site/species and light on O_2_ flux, ΔpH and [CO_3_^2-^]_S_:[CO_3_^2-^]_B_, a nonparametric test for repeated measures data in factorial designs was conducted in RStudio (version 0.98.1087) using the nparLD package (nonparametric analysis of longitudinal data in factorial experiments). This test was appropriate because more than one group of subjects (groups = species) was observed repeatedly over time (Noguchi et al., 2012). Light and dark profiles were measured on the same individual at the same location at different time points. Results of the ANOVA-type statistic are reported. To determine if surface [CO_3_^2-^] and [O_2_] differed from bulk [CO_3_^2-^] and [O_2_], independent t-tests were conducted separately for each response variable for each species and light condition.

## Results

### Oxygen, pH, and CO_3_^2-^ Microprofiles

**FIGURE 2:**
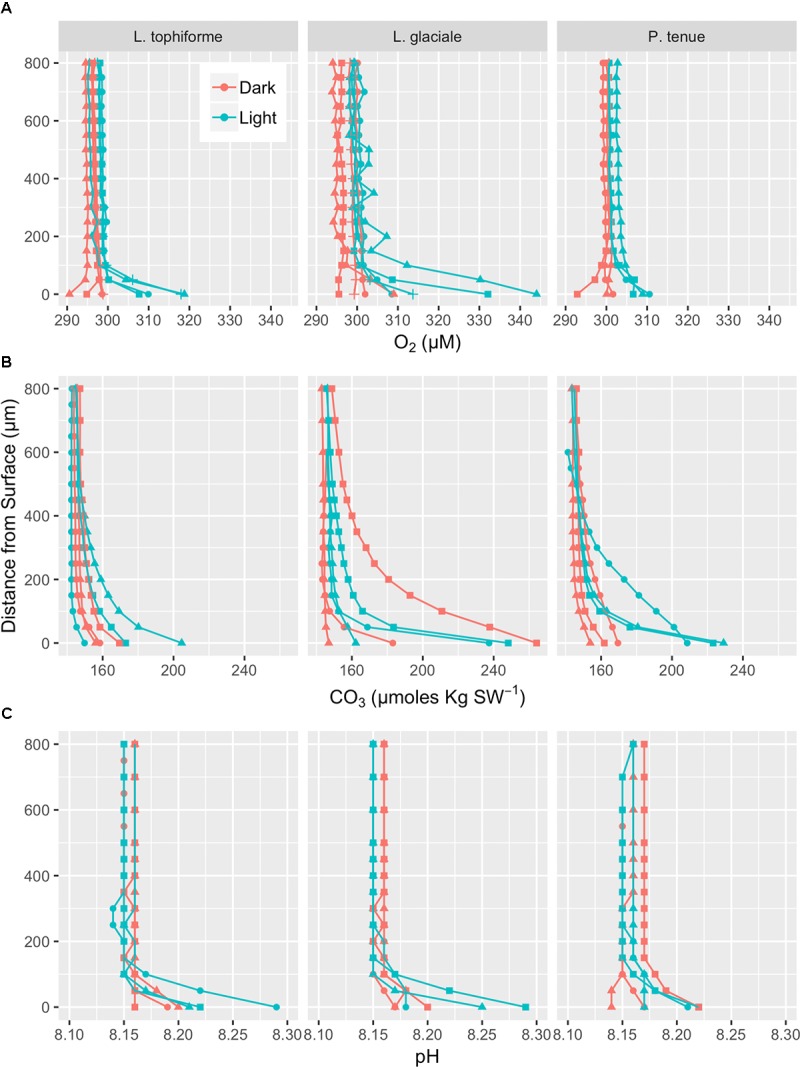
Microprofiles of oxygen concentration **(A)**, carbonate ion concentration **(B)**, and pH **(C)** measured with microsensors under saturating light (blue) and dark (red) conditions. Each profile is an average of four replicate profiles ± SE. The symbols represent measurements conducted on different individual algae.

**FIGURE 3:**
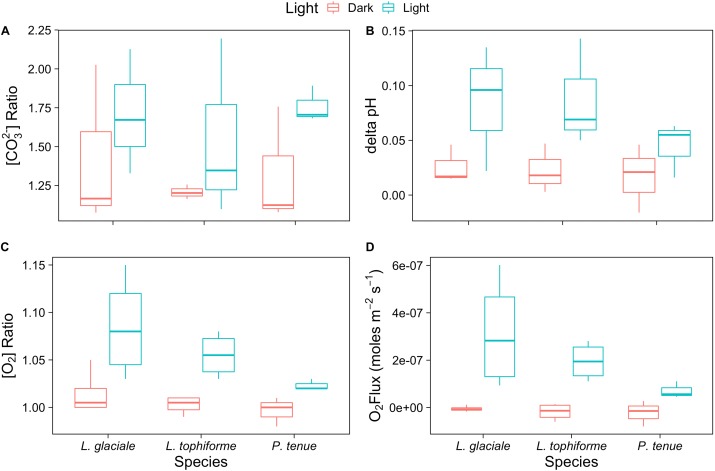
Boxplot of **(A)** carbonate ion concentration ratios (surface:bulk), **(B)** delta pH, and **(C)** oxygen concentration ratios at the algal surface to the concentration in the bulk seawater under saturating light and dark conditions for the coralline algae investigated. **(D)** Oxygen fluxes at the surface of the algae under saturating light and dark conditions.

Microprofiles of O_2_, CO_3_^2-^, and pH showed that despite reduced O_2_ concentrations due to respiration in the dark, surface pH, and CO_3_^2-^ concentrations both remained higher than the bulk seawater under both light and dark conditions (**Figures 2A–C**). This was observed on all three algal species. These trends are further shown by the ratio of [CO_3_^2-^]_S_:[CO_3_^2-^]_B_ and the delta pH (**Figures 3A,B**). There was no significant effect of site/species on either oxygen flux, ΔpH or [CO_3_^2-^]_S_:[CO_3_^2-^]_B_, but all parameters were significantly higher in the light than in the dark (O_2_ flux: ANOVA-type statistic (ATS) = 62.5, *p* = 2.7e - 15 ΔpH: ATS = 18.5, *p* = 1.7e-5; [CO_3_^2-^]_S_:[CO_3_^2-^]_B_: ATS = 5.9, *p* = 0.01; see Statistical Analysis above).

The O_2_ concentration at the surface was not significantly different from that of seawater in the dark, indicating that respiration rates (oxygen flux in the dark) were very low (**Figure 3C** and **Supplementary Table S2**). In the light, the surface concentration of O_2_ significantly increased due to photosynthesis and a small efflux into the bulk seawater was observed (**Figure 3D** and **Supplementary Table S2**).

### Surface Carbonate Chemistry

**FIGURE 4:**
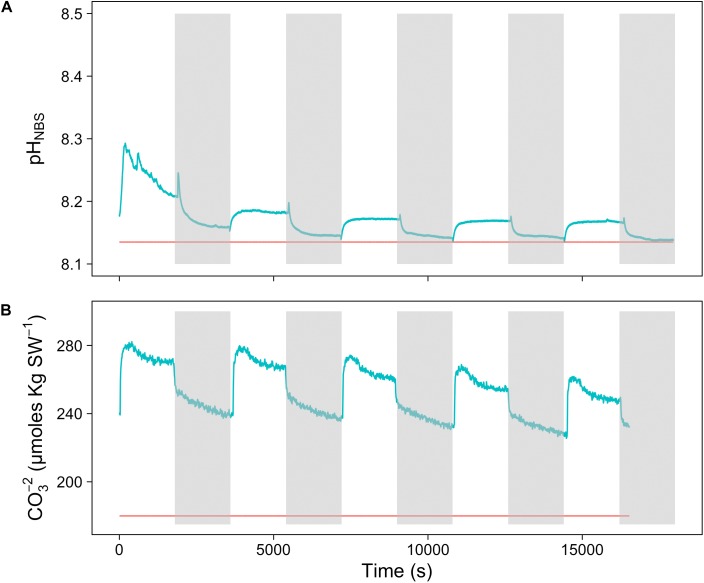
Surface **(A)** carbonate ion concentration and **(B)** pH of a *L. glaciale* rhodolith from Købbefjord measured in 30-min light/dark intervals. The gray shaded areas indicate periods of darkness. The blue lines represent values measured at the surface of the alga, and the red lines indicate bulk seawater concentrations.

**FIGURE 5:**
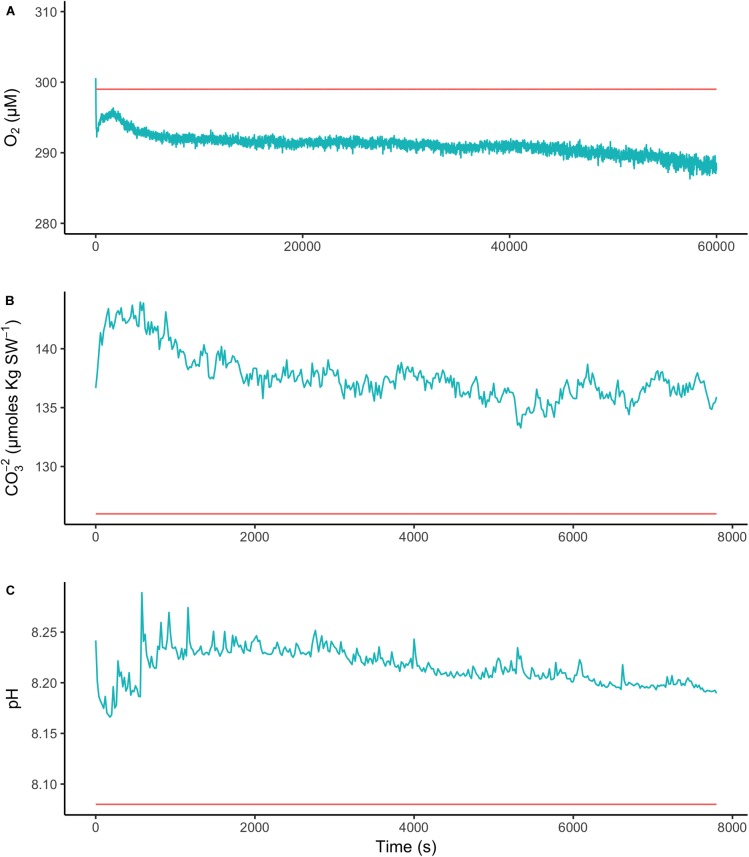
Surface (blue) and bulk seawater (red) concentrations of **(A)** oxygen, **(B)** carbonate ion, and **(C)** pH measured during a period of extended darkness on a *P. tenue* individual from Spitsbergen. Note that time axis for oxygen is longer than for pH and carbonate.

**FIGURE 6:**
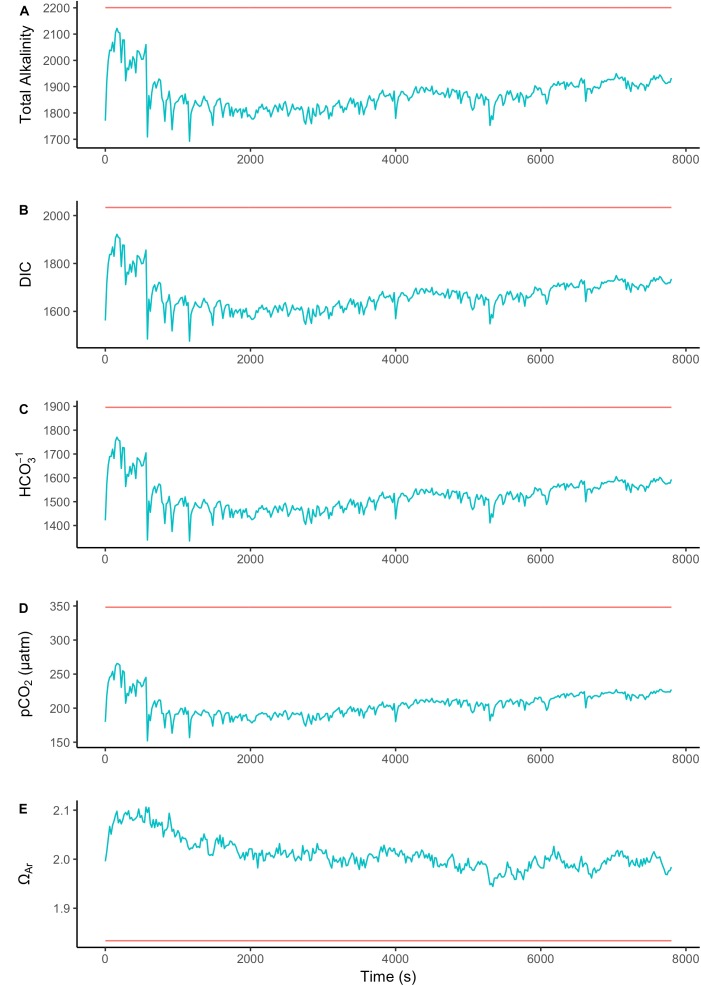
Surface (blue) and bulk seawater (red) concentrations of **(A)** total alkalinity (TA), **(B)** total dissolved inorganic carbon (DIC), **(C)** bicarbonate (HCO_3_^-^), **(D)** pCO_2_, and **(E)** aragonite saturation state calculated from the values measured in **Figure 5**. The units for TA, DIC, and HCO_3_^-^ are in μmoles Kg SW^-1^.

To assess the effect of light and darkness on the surface chemistry, we measured the dynamics of surface pH and [CO_3_^2-^] in response to saturating light and darkness. Both surface pH and [CO_3_^2-^] increased in the light and decreased in the dark at the surface of *L. glaciale* from Købbe Fjord (**Figure 4**). Upon illumination, the [CO_3_^2-^]_S_ increased sharply, and then slowly decreased before reaching steady state at a level that always remained above the levels in darkness (**Figure 4**). However, after darkening, the pH slightly increased before beginning to decrease and reaching a steady state. The opposite pattern was seen when the light was switched on, showing a slight dip in pH before it started increasing, and reaching a steady state. Regardless of whether the alga was in the light or dark, the pH_S_, and [CO_3_^2-^]_S_ always remained above the bulk seawater levels. Similar trends in light/dark dynamics were found for *P. tenue* and *L. tophiforme* (**Supplementary Figures S1**, **S2**). In order to determine when the [O_2_]_S_, pH_S_, and [CO_3_^2-^]_S_ reached steady state in the dark and to determine if they reach levels below the bulk seawater at any point, we monitored each parameter in the dark for up to 15 h. In *P. tenue* from Spitsbergen, the pH_S_ never dropped below pH_B_ in the dark, and the [CO_3_^2-^]_S_ remained above [CO_3_^2-^]_B_ for over 13 h, despite lower [O_2_]_S_ compared to [O_2_]_B_ (**Figure 5**). Similar patterns were found during extended periods of darkness for additional individuals of *P. tenue* and for *L. tophiforme* (**Supplementary Figures S3**, **S4**), although the pH_S_ dipped slightly below the pH_B_ in one specimen of *P. tenue* after 7 h (**Supplementary Figure S3**). From the pH and CO_3_^2-^, we calculated the remaining parameters of the carbonate system at the algal surface and in the bulk seawater using the package seacarb in R (see Materials and Methods). **Figure 6** shows that the surface DIC, pCO_2_, HCO_3_^-^, and TA remained lower than the bulk seawater, while the aragonite saturation state remained higher than the bulk seawater.

### Inhibition of Carbonic Anhydrase

The addition of AZ resulted in a slight decrease in surface O_2_ and in surface pH of *L. glaciale* (**Supplementary Figure S5**). The inhibition of carbonic anhydrase only inhibited delta pH at 85 μmol photons m^-2^ s^-1^ (**Supplementary Figure S6**), which was below light saturation. The light at which gross photosynthesis was saturating before AZ addition was 130 μmol photons m^-2^ s^-1^ (**Supplementary Figure S7**). Carbonic anhydrase inhibition resulted in the inhibition of gross photosynthesis, which showed different patterns depending on light intensity. CA inhibition was constant above 10 μmol photons m^-2^ s^-1^ at approximately 50–75%. Only at the lowest light level (2 μmol photons m^-2^ s^-1^), no inhibition was observed. This indicates that at only very low light intensities, the supply of CO_2_ by transport from the seawater (13 μM) and non-catalyzed conversion of HCO_3_^-^ is sufficient. However, at higher light intensities, and thus at higher photosynthetic capacity, CO_2_ supply from the seawater limits the photosynthetic rate, and CA is needed to enhance the CO_2_ supply (**Supplementary Figure S7**).

## Discussion

Our results show the carbonate chemistry conditions in the microenvironment of Arctic coralline algae. We did not observe strong differences in pH_S_ or [CO_3_^2-^]_S_ between the three species, indicating that in this case, the rhodolith versus crustose morphology did not have a strong influence on surface chemistry. Also, it suggest that our observations may hold generally for Arctic rhodoliths. The saturation state of calcite and aragonite remained greater than 1 under all conditions, despite respiration in the dark. Dark dissolution did not likely occur under our experimental conditions, even after hours of darkness, because TA at the algal surface was lower than in the bulk seawater, suggesting that dissolution was not the source of elevated CO_3_^2-^ ions. The lower alkalinity at the surface in the dark could be due to nutrient uptake, as the uptake of phosphate and ammonium ions results in a decrease in alkalinity (Wolf-Gladrow et al., 2007). These results support the growing evidence that coralline algae have strong biotic control over their skeletal structure (Kamenos et al., 2013, 2016; Nash et al., 2015) and microenvironments where active calcification is occurring (Hofmann et al., 2016; Cornwall et al., 2017).

Arctic rhodoliths and crustose coralline algae control their surface chemistry actively. The surface pH dynamics upon illumination and darkening were rapid (within seconds), showing the presence of a light-driven proton pump. Further evidence for the presence of a proton pump was provided by the inhibition experiment with *L. glaciale*, which showed that despite inhibition of gross photosynthesis, there was no significant change in delta pH. Therefore, a process independent of photosynthesis also influences the surface pH. Our observation differs from the recently proposed mechanism (Chrachri et al., 2018) in the marine diatom *Odontella sinensis*, whose surface pH was strongly inhibited by AZ, suggesting that surface pH is directly dependent on the rate of photosynthesis in this organism. Although photosynthesis in *L. glaciale* is strongly dependent on CA, it has an additional mechanism, a light-induced proton pump, that influences surface pH independently from photosynthesis. The same result has been reported for a tropical crustose coralline alga (Hofmann et al., 2016) and the calcifying green alga *Halimeda discoidea* (de Beer and Larkum, 2001). This photosynthesis independent, light-triggered proton pump may have been responsible for the rapid pH spikes we observed after turning off the light.

The surface carbonate ion dynamics were slower than the pH dynamics, suggesting that the carbonate dynamics were not only driven by pH. Because carbonate and bicarbonate are in almost instant equilibrium, the peaks in the carbonate dynamics likely represent rapid bicarbonate uptake by a carbon concentrating mechanism before a steady state is reached. The presence of a carbon concentrating mechanism in these algae has been recently supported by a separate study (Hofmann and Heesch, 2017). Additional support for the presence of a carbon concentrating mechanism utilizing HCO_3_^-^/H^+^ co-transport was provided by our measurements during extended darkness. Despite the higher surface pH and CO_3_^2-^ that we measured, the calculated TA at the algal surface during extended darkness was lower than the bulk seawater. This was explained by a strong reduction (ca. 75%) in the concentration of surface HCO_3_^-^, which was comparable to the reduction in protons (ca. 69%) associated with the elevated surface pH. Further evidence of HCO_3_^-^ uptake can be provided by modeling the seawater carbonate system. If we assume a photosynthetic quotient (O_2_ released: CO_2_ fixed) of 1 (but note that Chisholm (2000) reported a photosynthetic quotient of 1.05–1.48 for tropical coralline alga), we would expect the surface DIC concentration to decrease the same magnitude as the surface O_2_ concentration increased. On average, the O_2_ concentration at the surface increased 10 μM under saturating light. Therefore, if we consider the bulk seawater has a pH of 8.15 and TA of 2200, the [CO_3_^2-^] is 139 μM. An increase in pH to 8.2, which we observed, accompanied by a 10 μM decrease in DIC as CO_2_ would result in a [CO_3_^2-^] of 50 μM. On the other hand, a decrease in DIC as HCO_3_^-^ would result in a [CO_3_^2-^] of 159 μM, which is comparable to the concentrations we observed at the surface of all the coralline algae investigated. Therefore, it is highly likely that the changes in surface chemistry are due to HCO_3_^-^ uptake coupled to H^+^ co-transport or OH^-^ exchange. Our data, along with those from Comeau et al. (2013) and Hofmann et al. (2016) support the hypothesis by Borowitzka and Larkum (1987) that there is a H^+^/HCO_3_^-^ symporter or HCO_3_^-^/OH^-^ antiporter in coralline algae. Co-transport of HCO_3_^-^ and protons away from the calcifying surface and into the cell results in optimal conditions for calcification at the cell surface under both light and dark conditions, and elevated HCO_3_^-^ concentrations inside the cell neutralize the protons, providing an inorganic carbon source for photosynthesis in the light. This mechanism is in stark contrast to the mechanism proposed in some non-calcifying macroalgae, Characean algae, and freshwater flowering plants, where HCO_3_^-^ use is coupled to H^+^ extrusion, producing acidic zones where the concentration of CO_2_ is elevated due to the low pH and therefore diffuses into the cell (Moulin et al., 2011; Raven and Hurd, 2012). The H^+^ extrusion must be accompanied by OH^-^ extrusion in alkaline zones. Our microsensor measurements do not support the presence of acidic or alkaline zones in coralline algae, although these zones are hypothesized to be much smaller than the diameter of a microsensor, and therefore cannot be accessed. Nevertheless, our results strongly suggest that HCO_3_^-^ is taken up directly, with either a net H^+^ co-transport or net OH^-^ efflux, which results in a net increase in surface pH.

While this mechanism is easily explained under light conditions, it remains to be determined why and how Arctic coralline algae maintain elevated pH_S_ and [CO_3_^2-^]_S_ in darkness. We hypothesize that these observations are a result of high rates of light independent carbon fixation, which is a common adaptation in polar macroalgae that facilitates their survival through the polar winter (Wiencke et al., 2009). Light independent carbon fixation (or β-carboxylation) is an anaplerotic pathway that involves the enzymes phosphoenol pyruvate carboxylase (PEPC) or phosphoenol pyruvate carboxykinase (PEPCK) to fix carbon dioxide (or bicarbonate) to the β-carbon of PEP or pyruvate. This process reduces carbon loss under darkness and produces important metabolites, such as amino acids. High rates of light independent carbon fixation would explain the continued inorganic carbon uptake (in this case as HCO_3_^-^) and elevated surface pH in the dark, despite oxygen consumption resulting from dark respiration. Further evidence for this hypothesis can be seen in the data from Hofmann and Heesch (2017), who reported the total and organic ^13^C:^12^C fractionation in Atlantic rhodoliths. The organic fraction ∂^13^C signatures of Arctic rhodoliths, including specimens from the same collection sites used in this study, deviated from the linear trend with latitude. The Arctic rhodoliths had organic material more enriched in ^13^C than would be expected based on the latitudinal trend, which may be due to higher rates of anaplerotic reactions that result in ^13^C enriched carboxyl carbons (Savidge and Blair, 2004). Although the CO_2_ concentration at the algal surface (based on the measured pH and CO_3_^2-^ concentrations) was lower than the bulk seawater and hence suggests CO_2_ uptake by the alga in the dark, our hypothesis is at this time speculative, as we did not directly measure CO_2_ uptake in the dark. Additional possibilities for dark CO_2_ uptake [e.g., carbamoyl phosphate synthase (Raven et al., 1990)] cannot be excluded. Further studies measuring direct CO_2_ fluxes would be necessary to confirm our hypothesis.

## Conclusion

In conclusion, our results suggest that Arctic corallines have strong biological control over their surface chemistry, where active calcification occurs. High rates of light independent carbon fixation are likely an adaptive strategy in Arctic coralline algae for limiting carbon loss during long periods of darkness, and additionally create a microenvironment that prevents dissolution in the dark. Our results suggest that net dissolution in the dark is not currently a threat to Arctic coralline algae during the summer. However, long exposure to darkness resulted in a surface pH below seawater pH, suggesting that dissolution could still occur during winter. These unique physiological mechanisms likely play an important role in facilitating the survival of Arctic coralline algae under harsh conditions, but further studies will be needed to determine if these mechanisms could facilitate adaptation to ocean acidification in the future, which would have important implications for Arctic marine ecosystems.

## Data Availability

The datasets generated during and/or analyzed during the current study are available from the corresponding author upon reasonable request.

## Author Contributions

LH designed and carried out the experiment and wrote the manuscript. KS collected the samples from Greenland and edited the manuscript. DdB edited the manuscript.

## Conflict of Interest Statement

The authors declare that the research was conducted in the absence of any commercial or financial relationships that could be construed as a potential conflict of interest.

## Supplementary Material

FIGURE S1 Light/dark dynamics of [CO_3_^2-^] in *P. tenue*.

FIGURE S2 Light/dark dynamics and extended darkness [CO_3_^2-^] in *L. tophiforme*.

FIGURE S3 Surface pH and [O_2_] of *P. tenue* during extended darkness.

FIGURE S4 Surface [CO_3_^2-^] and pH of *L. tophiforme* during extended darkness.

FIGURE S5 Surface [O_2_] and pH before and after AZ addition.

FIGURE S6 Delta pH at the surface of *L. glaciale* before and after AZ addition.

FIGURE S7 Effect of AZ on gross photosynthesis in *L. glaciale*.

TABLE S1 Thickness of the diffusional boundary layer at different locations on the thalli.

TABLE S2 *T*-tests comparing surface and bulk [O_2_] for each species.
