# Supplementary material for: Arctic Coralline Algae Elevate Surface pH and Carbonate in the Dark
Source: Front Plant Sci. 2018 Sep 25;9:1416. doi: 10.3389/fpls.2018.01416 (PMC6167962; doi:10.3389/fpls.2018.01416)
Supplement: FIGURE S1 — Light/dark dynamics of [CO32-] in P. tenue. [file Table_1.DOCX]

Supplementary Material

**Arctic coralline algae elevate surface pH and carbonate in the dark**

**Laurie Carol Hofmann*, Kathryn Schoenrock and Dirk de Beer**

***Correspondence:** Corresponding author: lhofmann@mpi-bremen.de

The following Supporting Information is available for this article:

Table S1. Thickness of the diffusional boundary layer at different locations on the thalli

Table S2. T-tests comparing surface and bulk [O_2_] for each species

Fig. S1. Light/dark dynamics of [CO_3_^2-^] in *P. tenue*

Fig. S2. Light/dark dynamics and extended darkness [CO_3_^2-^] in *L. tophiforme*

Fig. S3. Surface pH and [O_2_] of *P. tenue* during extended darkness

Fig. S4. Surface [CO_3_^2-^] and pH of *L. tophiforme* during extended darkness

Fig. S5. Surface [O_2_] and pH before and after AZ addition

Fig. S6. Delta pH at the surface of *L. glaciale* before and after AZ addition

Fig. S7. Effect of AZ on gross photosynthesis in *L. glaciale*

**Supplementary Table 1. Thickness of the diffusional boundary layer** (DBL) of each species from each site under saturating light and dark conditions. The DBL was measured at the base and tip of branches of *L. glaciale* to show the importance of standardizing the location of measurements for comparison. Values are based on pH microprofiles and are means ± standard deviation (SD).

| Species | Base/Tip | Light/Dark | DBL (µm) |
| --- | --- | --- | --- |
| *L. glaciale* | Base | Light | 187 ± 17 |
|  | Base | Dark | 157 ± 3 |
|  | Tip | Light | 108 ± 3 |
|  | Tip | Dark | 107 ± 4 |
| *L. tophiforme* | Tip | Light | 87 ± 24 |
|  | Tip | Dark | 87 ± 17 |
| *P. tenue* | Tip | Light | 127 ± 28 |
|  | Tip | Dark | 153 ± 62 |

**Supplementary Table 2.** **Results of independent t-tests** comparing surface and bulk oxygen concentrations of algae from Greenland (Akia Peninsula and Købbefjord) and Spitsbergen. Significant differences are highlighted in bold.

|  | Light | Dark |
| --- | --- | --- |
| Akia | **t = 5.8, df = 3.2, p = 0.009** | t = 0, df = 3.3, p = 1 |
| Købbe | **t = 3.1, df = 3.03, p = 0.05** | t = 1.4, df = 3.9, p = 0.2 |
| Kongsjord | **t = 6.4, df = 2.3, p = 0.02** | t = -0.5, df = 2.06, p = 0.7 |


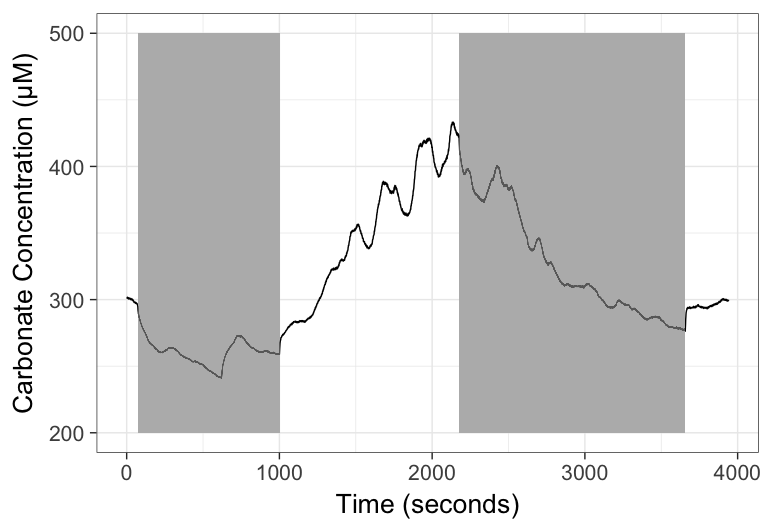


**Supplementary Figure 1. Surface carbonate ion concentrations of *P. tenue*** from Spitsbergen under intervals of dark (shaded) and saturating light (unshaded) periods.


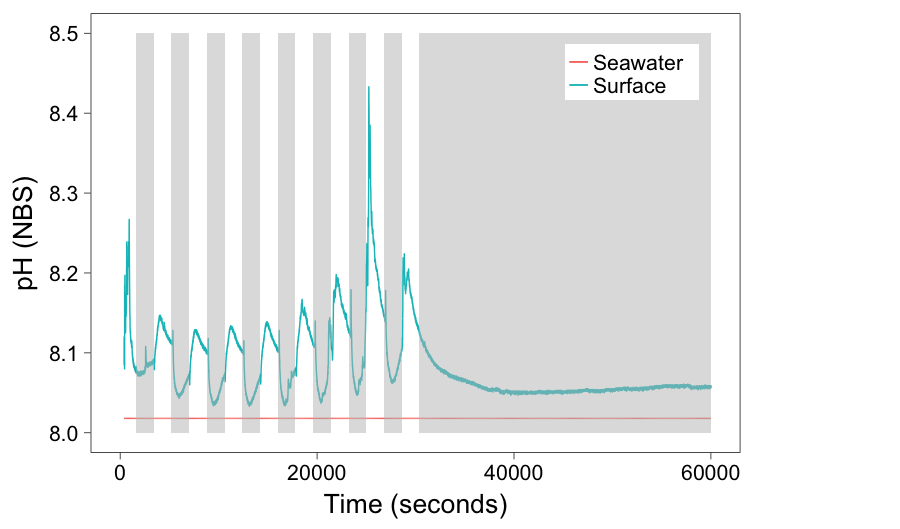


**Supplementary Figure 2. Surface (blue) and bulk seawater (red) pH of *L. tophiforme*** from Akia Peninsula during short intervals of saturating light (white background) and darkness (shaded grey background) and an extended period of darkness.


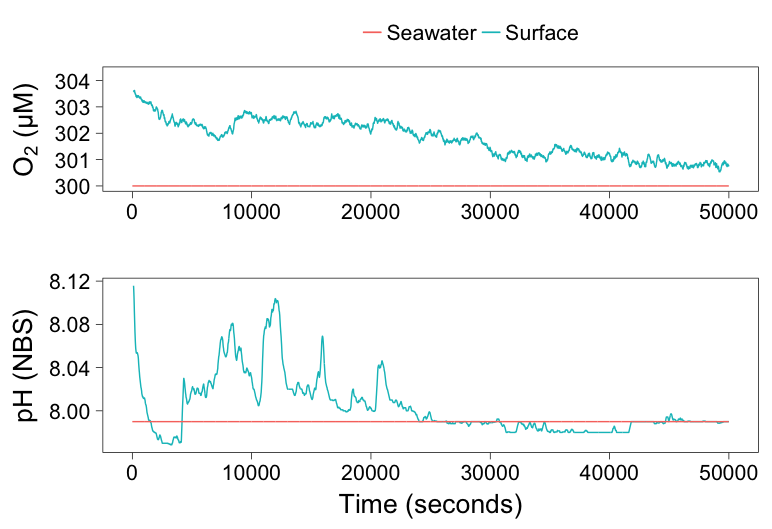


**Supplementary Figure 3.** **The surface oxygen (top panel) and pH (bottom panel) of *P. tenue*** from Spitsbergen during extended darkness. Time 0 indicates the point at which the light was turned off.


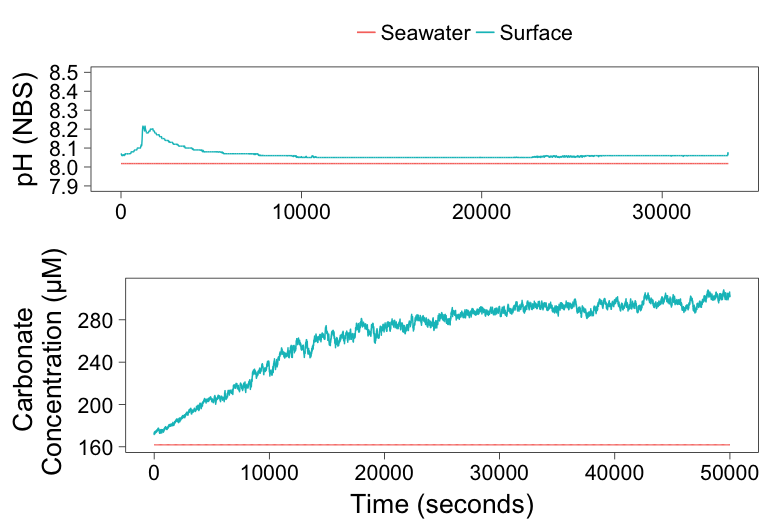


**Supplementary Figure 4. Surface (blue) and bulk seawater (red) pH (top panel) and carbonate ion** concentration (bottom panel) of *L. tophiforme* from Akia Peninsula during an extended period of darkness.


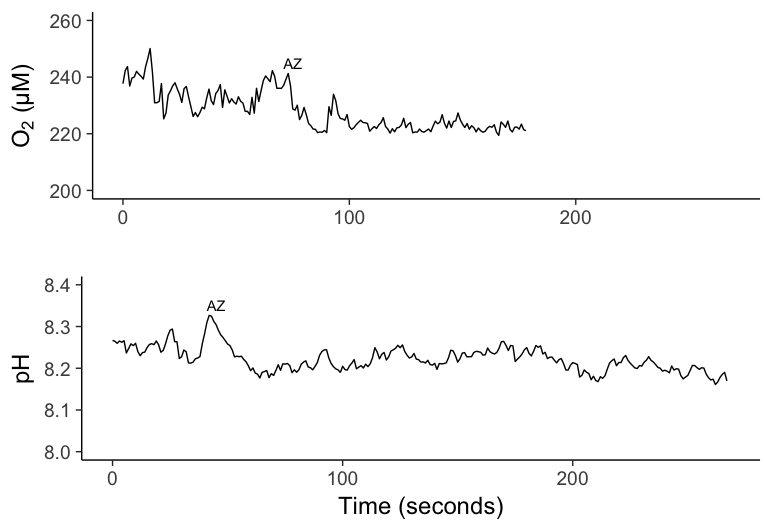


**Supplementary Figure 5. Surface oxygen (top) and pH (bottom) values** recorded before and after the addition of the carbonic anhydrase inhibitor acetazolamide (AZ). The symbol “AZ” on each graph represents when AZ was added to the reservoir.


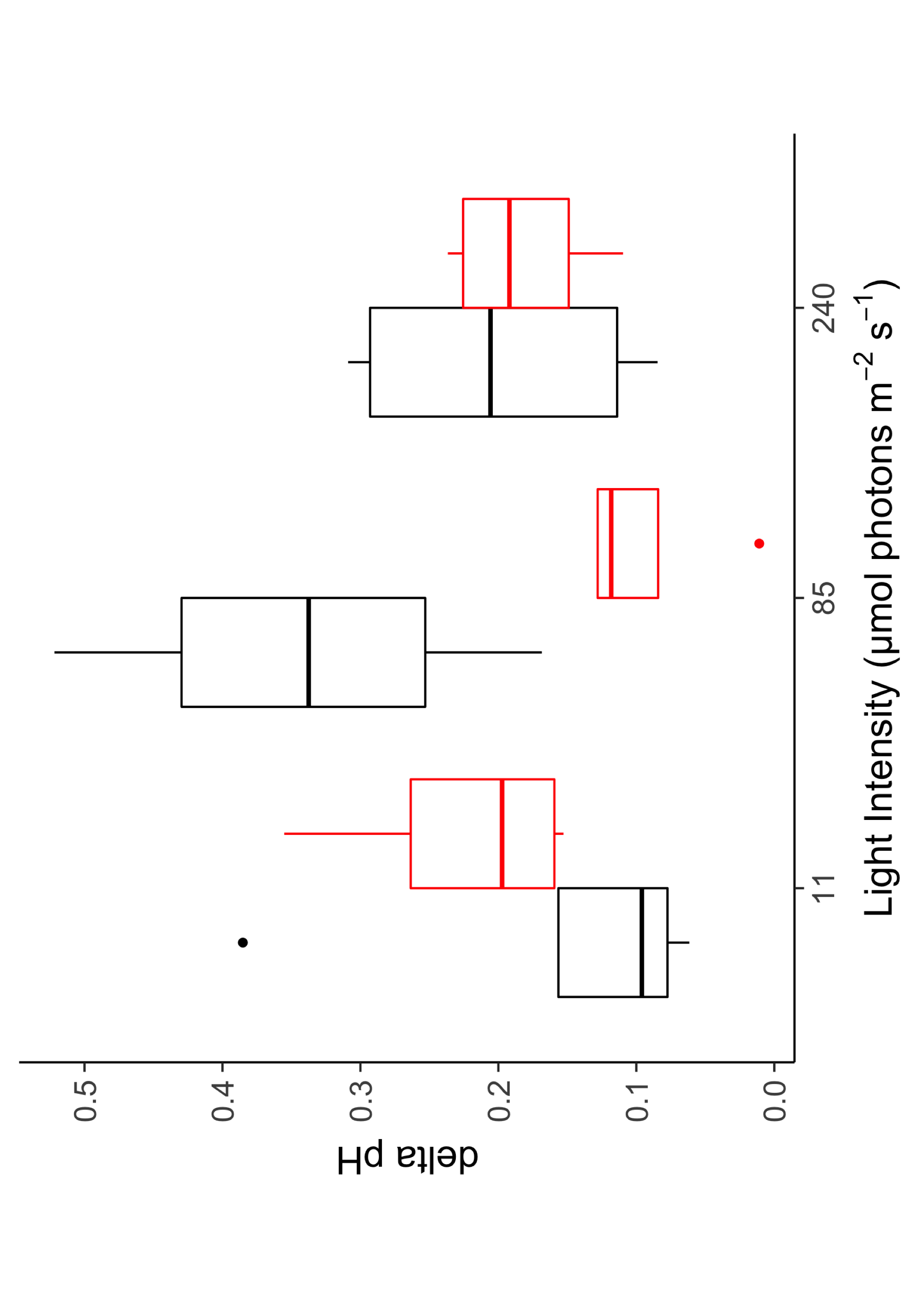


**Supplementary Figure 6.** **The change in pH** (surface – bulk seawater) measured before (black) and after (red) the addition of the external carbonic anhydrase inhibitor acetazolamide to *L. glaciale*.

**
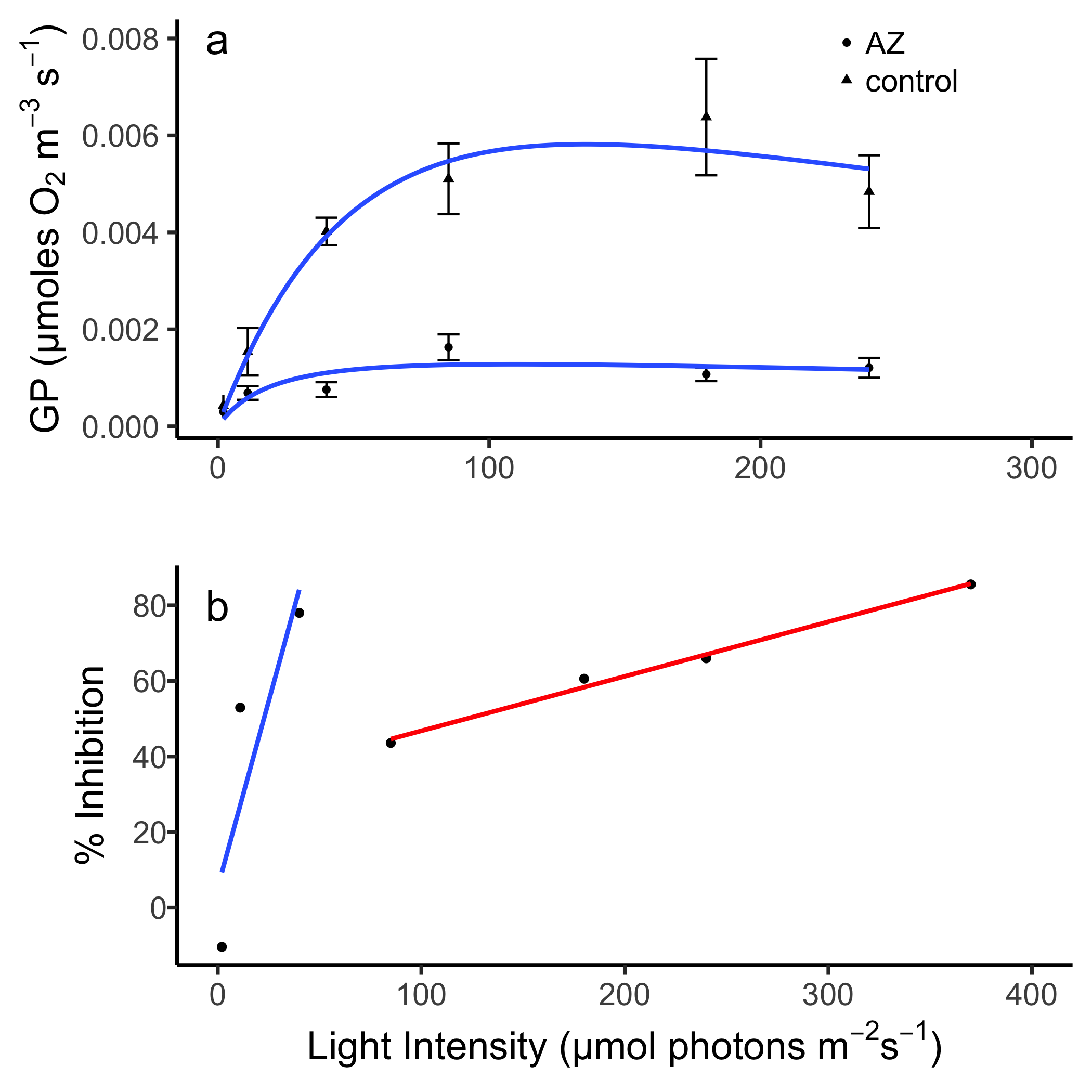
**

**Supplementary Figure 7.** **a) Gross photosynthesis** **versus irradiance** light curve for *L. glaciale* before (triangles) and after (circles) the addition of the external carbonic anhydrase inhibitor, acetazolamide, and b) the percent of inhibition of gross photosynthesis under limiting (blue line) and saturating (red line) light intensities.
